# Tumor–stroma ratio in colitis-associated colorectal cancer

**DOI:** 10.48101/ujms.v130.13250

**Published:** 2026-01-02

**Authors:** Kajsa Björner, Miklos Gulyas, Per M. Hellström, Dominic-Luc Webb

**Affiliations:** a Department of Medical Sciences, Gastroenterology and Hepatology Section, Uppsala University, Uppsala, Sweden; b Department of Immunology, Genetics and Pathology, Uppsala University, Uppsala, Sweden; c DLW Bioanalytics, Märsta, Sweden

**Keywords:** Tumor microenvironment, oncogenesis, colorectal cancer, colitis-associated colorectal cancer, inflammatory bowel disease, tumor biology

## Abstract

High stroma content, as measured by tumor-stroma ratio (TSR), is generally a negative prognostic parameter for epithelial cancers, including sporadic colorectal cancer (sCRC). Inflammatory bowel disease patients have higher risk for colorectal cancer than the background population. Evidence suggests that this colitis-associated colorectal cancer (CAC) is more aggressive and occurs at younger age than sCRC. CAC also differs from sCRC in oncogenesis and prognosis. This study tests the hypothesis that TSR in CAC tumors correlates with survival. Age at CAC diagnosis relative to TSR was also explored. TSR was quantified in 36 CAC cases. In routine hematoxylin–eosin staining, the amount of stroma was estimated in categorical steps of 10% increments per image field. The area with highest amount of stroma and tumor tissue at all quadrants of the visual field boundary was scored for TSR. For statistical analysis, tumors were divided into stroma-high (> 50%) or stroma-low (≤ 50%). Of all cases, 22 were stroma-high and 14 were stroma-low. Five-year survival in the stroma-high group was 32% (*n* = 22), compared to 71% (*n* = 14) in the stroma-low group (*p* = 0.049). High stroma content was more frequent if cancer diagnosis was before 60 years of age (17/23) compared to after 60 years of age (5/13). Despite differences in oncogenesis and tumor biology in CAC compared to sCRC, high stroma content also predicts worse outcome in CAC and is particularly common in younger patients.

## Background

### Colitis-associated cancer

Inflammatory bowel disease (IBD) has increased in Sweden over the last century. IBD is subdivided into ulcerative colitis (UC), Crohn’s disease (CD), and unclassified inflammatory bowel disease (IBD-U). IBD is associated with increased risk of colorectal cancer (CRC) (1–4), making this patient group of particular concern. Elevated risk of colitis-associated colorectal cancer (CAC) has been associated with young age at diagnosis, long duration of IBD, extensive disease, active inflammation, and primary sclerosing cholangitis (5–8).

### Stroma in epithelial cancer

Stroma is the supportive tissue of an epithelial organ or tumor, consisting of connective tissue and vessels surrounding epithelial structures. Tumor cells and tissue stroma evolve together. In oncogenesis, when epithelial cells develop into malignant cancerous tissue, a crosstalk between epithelial cells and surrounding stroma develops (9). This is seen in extracellular matrix, macrophages, and fibroblasts. Fibroblasts progress to cancer-associated fibroblasts (CAF) after activation from cancer cells (10). One difference in the tumor microenvironment of CRC compared to other tumors is the microbiome that affects epithelial surface and stroma (11). Tumor–stroma, mainly through CAFs, stimulate cancer cell proliferation, remodeling of extracellular matrix, and angiogenesis (10). Tumor–stroma regulates cancer cells and can be involved in tumorigenesis by inducing stem cell-like properties or epithelial to mesenchymal transition (12).

Cancer treatment has historically been based on clinicopathological characteristics, such as, patient age and performance status, tumor type, differentiation, tissue infiltration, and presence of metastasis (13). The tumor-node-metastasis (TNM) classification is used in clinical practice to choose the most suitable treatment for the individual patient (14) and is supplemented with gene analysis and microsatellite instability status (15, 16). Still, in node-negative colon cancer, a group with generally good prognosis, some develop disease recurrence; determining both tumor–stroma ratio (TSR) and BRAF (B-Raf proto-oncogene, serine/threonine kinase) mutations might help in prognostication (17). Certain pathological features of the tumor can be just as important as biomarkers of disease progression. Tumor budding and the tumor stroma are features that can be exploited to histologically evaluate risk of metastasis (18). TSR has been reported to be a strong prognostic indicator in sporadic colorectal cancer (sCRC) (19–22) and has recently been validated in a prospective multicenter study (23). TSR has a good interobserver validation and reproducibility (24). Carcinogenesis differs in CAC compared to sCRC in that CAC involves the inflammation–dysplasia–carcinoma pathway, rather than the canonical adenoma–carcinoma pathway, to manifest cancer development (6, 25, 26) and worsened survival (4).

Four Consensus Molecular Subtypes (CMS) have been established by the International Colorectal Cancer Subtyping Consortium (27). The four CMS are CMS1 (microsatellite-instability, immune), CMS2 (epithelial, canonical), CMS3 (metabolic), and CMS4 (mesenchymal). CMS4 is known to have worse prognosis (27) and has been indicated to be more common in CAC (28).

CAC has a different age profile than sCRC, with higher cancer risk in younger IBD patients (29–31). There has been mixed evidence as to whether CAC is more aggressive than CRC (32). Some of the largest studies exploring this fact found survival to be lower in CAC than CRC regardless of UC or CD background (33). Recent Swedish data indicate that IBD negatively influences prognosis of colon cancer, but not rectal cancer, after the first year of disease (34, 35). The difference in survival between CAC and sCRC is likely driven by patients < 65 years of age (36). We hypothesized that stroma-high tumors might be more common in IBD patients diagnosed with cancer earlier in life as a result of a colitis-driven oncogenesis that may be regarded as true CAC not present in the main population. IBD patients presumably retain similar risk of contracting sCRC through the adenoma–carcinoma pathway that increases throughout life. The primary aim of our present study was therefore to determine TSR in CAC patients and relate this to survival. TSR differences at different ages of CAC diagnosis were also explored. A secondary aim was to evaluate if TSR differs with age of onset of CAC.

## Methods

### Study subjects and samples

This study consisted of patients with IBD and CRC diagnosis between 1970 and 2020 identified from inpatient and outpatient registries in Uppsala region, Sweden. Inclusion criteria for CAC patients were: confirmed diagnosis of IBD; concomitant colorectal adenocarcinoma and with available blocks of cancer tissue. Exclusion criteria were; registry diagnosis of IBD or CRC that could not be confirmed in medical records. Out of totally 56 cases with a CAC primary tumor, 47 were surgical specimens and 9 were biopsies. The nine biopsy specimens were excluded due to insufficient material. Of the 47 surgical specimens, 36 could be evaluated for TSR; 11 were excluded due to prior chemo- or radiotherapy. Slides were chosen from the most invasive part of the tumor as indicated in the pathology report, if this was not clear from reports several slides were assessed and the one with highest percentage stroma was chosen.

To explore differences in TSR with age of cancer diagnosis, data were sorted with age cut-offs 50 and 60 years of age, which are reported age windows when incidence of sCRC rapidly increases (37, 38).

### Scoring

Microscopic evaluation was done according to the procedure of van Pelt et al. (39). After routine staining with hematoxylin–eosin solution, tissue sections of 4 µm thickness were analyzed by brightfield microscopy. Areas with high amount of stroma were selected with 5× magnification. The area with highest presence of stroma, but with tumor tissue at all quadrants within the visual field boundary, was selected and scored at 10× magnification. Amount of stroma was scored within nearest 10% increment (e.g. 10, 20, 30%, etc.) in the image field with the highest amount of stroma. Large vessel and necrotic areas were avoided, but if not possible, they did not contribute to the stroma estimate. One slide per patient was examined (i.e. one region of interest per patient); initially by KB and finally by MG, thus reaching consensus. Patient information was blinded at scoring.

### Statistics

For statistics, cases were sorted into stroma-high with > 50% stromal area and stroma-low with ≤ 50% stromal area in alignment with sCRC recommendations (39). Non-parametric tests were used for all statistical calculations with data presented as median and range. Survival rates were assessed using Kaplan–Meier plots with log-rank test with censoring at 60 month (i.e. 5 years). Decline in stroma-high versus stroma-low survival *p*-value with sample size was simulated with R programming language (40–42) by 10× expansion of original data (from *n* = 36 to *n* = 360) followed by random sub-sampling from *n* = 6 to *n* = 36 (details and source code in Supplement 1). Groups were compared with Mann–Whitney U-test. Statistical analyses were conducted using Statistica software, version 13 (TIBCO Software Inc., Palo Alto, CA, USA).

## Results

### Visual assessment of CAC tumor sections – large variation in TSR in CAC

**Figure 1:**
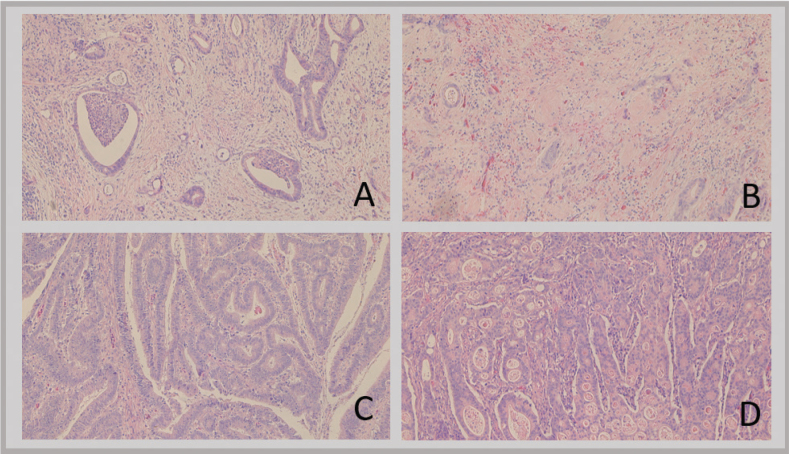
Examples of high and low stroma content in CAC. A and B exemplify high stroma. C and D exemplify low stroma. CAC: colitis-associated colorectal cancer.

Examples of high and low stroma content are depicted in Figure 1. In A and B there is mainly stroma interspersed with islets of tumor cells. C and D are tumor-rich and there is only sparse amount of stroma.

### Clinical characteristics in relation to TSR

Clinical characteristics of the examined patient group are presented in Table 1. The most common diagnosis was UC (*n* = 24). CD was about half of this (*n* = 11). The remaining case was IBD-U. There were more males (*n* = 23) than females (*n* = 13). Even though male cases had numerically more stroma-high ratios, no statistical difference was seen (*p* = 0.26). Regarding extension and location of IBD, the majority (24 out of 36 cases) had extensive colitis, followed by ileo-cecal location (5 out of 36). Numerically, the main location of cancer was the right colon (18), followed by the rectum (7). There were more patients at stage 4 in those excluded due to insufficient material.

**Table 1:** Patient characteristics.

Parameter	All	Stroma high	Stroma low	
*N*	36	22	14	
**Sex**				*p* ^§^
Female	13 (36%)	6	7	0.26
Male	23 (64%)	16	7	
**Diagnosis**				
CD	11	7	4	
UC	24	14	10	
IBD-U	1	1	−	
**Age at diagnosis**				*p* ^*^
**IBD** Median (Range)	25.5 (3–86)	23.5 (11–66)	30.5 (3–86)	0.51
**Cancer** Median (Range)	51 (17–95)	44 (21–82)	67 (17–95)	0.09
**Stage at cancer diagnosis**				
Stage 1	7	4	3	
Stage 2	14	8	6	
Stage 3	14	9	5	
Stage 4	1	1	−	
**Extension of IBD**				
Proctitis	3	1	2	
Left-sided	3	2	1	
Extensive	24	13	11	
ileocecal	5	5	−	
Unknown extent	1	1	−	
**Localization of cancer**				
Rectum	7	6	1	
Left colon	6	4	2	
Right colon	18	10	8	
Multiple locations	3	1	2	
Anastomosis	2	1	1	

§ Presence of stroma high related to sex.

* Stroma-high vs stroma-low.

UC: ulcerative colitis; CD: Crohn’s disease; IBD-U: unclassified inflammatory bowel disease.

### High TSR predicts poor survival

**Figure 2:**
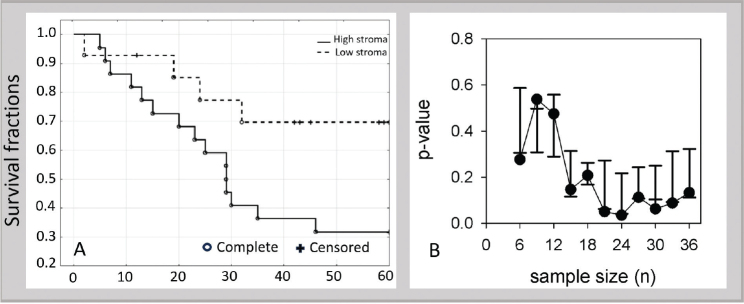
(A) Survival curve for CAC comparing stroma-high to stroma-low groups, log-rank test *p* = 0.049. (B) Simulated decline in *p*-value as a function of sample size revealing *p*-value reaches an essentially flat line by *n* = 36 (data are median and SEM (standard error of the mean) of seven *p*-values). CAC: colitis-associated colorectal cancer.

CRC survival of the stroma-high group was lower than for the stroma-low group by log-rank test (*p* = 0.049), Figure 2A. The 5-year CRC survival in the stroma-high group was 32% (7 of 22), compared to 71% (10 in 14) in the stroma-low group. A plot of decline in *p*-value with sample size using randomized sub-sampling from *n* = 6 to *n* = 36 revealed that *p*-value deflected to a flat line before *n* = 36 (piecewise two-segment breakpoint was *n* = 24, *p* = 0.002) (Figure 2B).

Median survival times could not be compared since more than half of the stroma-low group were still alive. Median follow-up time independent of survival status was 29 months (12 events, 55%) for the stroma-high (range 5–372 months) versus 44 months (5 events, 36%) (range 2–240 months) for the stroma-low group (*p* = 0.55).

### TSR related to age of onset of CAC

**Figure 3:**
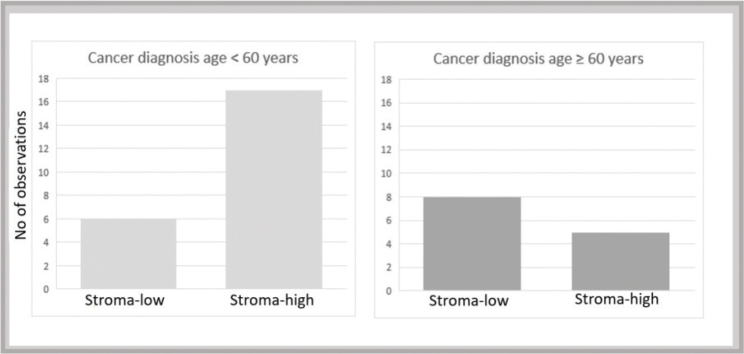
Stroma-high tumors occur more frequently in patients with cancer diagnosis < 60 years of age (Mann–Whitney, *p* = 0.041).

The median age for CAC diagnosis was 51 (range 17–95) years (Table 1). The stroma-high group was on average younger, 44 (21–82) years at cancer diagnosis than the stroma-low group, 67 (17–95) years (*p* = 0.09). There was no age difference at initial IBD diagnosis between the stroma-high and stroma-low groups (*p* = 0.51). Stratifying data by age using a cut-off of 50 years of age at CAC diagnosis, stroma-high tumors were numerically more frequent in those with CAC diagnosed before 50 years of age (12 of 16; 75%) compared to those diagnosed after age 50 (10 of 20; 50%) but not with statistical significance, *p* = 0.13. With a cut-off of 60 years of age at CAC diagnosis, stroma-high tumors were more frequent in patients diagnosed before age 60 years (17 of 23; 74%) compared to those diagnosed after age 60 (5 of 13; 38%) (*p* = 0.041, Figure 3).

## Discussion

As previously shown with sCRC, TSR appears to be a possible prognostic tool for CAC. A significant difference between stroma-high and stroma-low CACs was detected. The sample size of 36 combined with a threshold *p*-value indicates a low magnitude of effect at 5 years. The simulation in Figure 2B suggests that an increase in sample size, even a large increase, might have negligible effect on *p*-value. The *p*-value can be predicted to decline over time as a result of most stroma-low patients continuing to add follow-up months. However, after 5 years, factors such as deaths from other causes set additional constraints and introduce more non-informative censoring.

The TNM classification is the anatomical foundation for treatment decisions in all CRC. In recent years, prognostication has been sharpened with gene analysis and microsatellite instability evaluation, but further improvement is needed. With sharper prognostication tools, treatments can be better tailored to individual patients, as in decisions for adjuvant therapy.

The stroma-rich CMS4 has the highest mortality of the different CMS (27). That stroma-rich tumors have worse prognosis is often explained by Paget’s ‘Seed and Soil’ hypothesis. Stromal cells are considered to create a more favorable environment for the epithelial cancer cells at its origin as well as in metastases (43).

TSR assessment may be even more important for patients with CAC, Rajamäki *et al.* showed that CMS2 was under-represented in CAC, whereas CMS4 was more abundant (28). Proteomic analysis has revealed that CMS4 shows the most unique profile, revealing overexpression of proteins related to angiogenesis, extracellular matrix, and focal adhesion (44). This might explain why CAC has worse survival than sCRC. In most papers describing TSR in sCRC, there is a preponderance for the stroma-low group (21, 23, 45, 46). In our study, the stroma-high group comprised 61% of all cases, which aligns with the notion that stroma-rich tumors may be more common in CAC.

CAC diagnosed in patients over the age of 60 can be hypothesized to express features similar to sCRC. Younger IBD patients show a higher relative risk of CAC (29). Elderly IBD patients do not have a higher risk of CRC than their age-matched peers (4, 30). IBD onset at old age has been reported to not increase the risk of CAC (31, 47). This is in agreement with our present findings that patients below 60 years of age at cancer diagnosis have a higher occurrence of stroma-high tumors than those with diagnosed with CAC over 60 years of age. Exactly at what age the adenoma–carcinoma–pathway becomes more common than the inflammatory pathway is open to speculation.

Activation of CAFs has been attributed to several cytokines, among which are IL-1β (48) and iNOS (49). In our previous work, we showed high immunoreactivity of IL-1β as well as iNOS in the epithelial cells of CAC (50). In the stroma, IL-1β immunoreactivity was higher in CAC than in normal colon mucosa, whereas iNOS immunoreactivity was lower in CAC than in normal colon mucosa. This is in line with previous data of IL-1β being a biomarker of sCRC invasiveness (51, 52). Furthermore, increased NO-generation in a cell may select mutant p53-cells and contribute to tumor angiogenesis (44).

### Strengths and limitations

The strength of our primary observation lies in the fact that it highlights the importance of tumor stroma in CAC comparable to previous findings in sCRC. This study was also limited in follow-up time and possibly sample size. Statistical analyses of subgroups were not deemed meaningful. As with other studies, due to scarcity of CAC patients, subgroup analysis was under-powered. Since occurrence of CAC is a relatively rare complication of IBD, there is only a limited number of subjects spanning over a 40-year period to be evaluated for this research question. With improved surveillance and IBD treatments entering the market, occurrence and survival of CAC could change in the future. Our study reveals a signal that can readily be investigated in larger populations. Further subclassification of CAC should be possible for more accurate prognosis based on onset of malignant disease and also histological features of the cancer tissue. As with other prognostic factors, the present finding of high TSR as a negative prognostic factor must be validated in larger prospective studies.

## Conclusions

Stroma-rich tumors suggest worse prognoses. Those diagnosed with cancer at a younger age may be more likely to have high stroma content.

## Supplementary Material



## Data Availability

Images and data that are used in the results and conclusions in this study can be provided upon reasonable request.

## References

[1] Ekbom A, Helmick C, Zack M, Adami H-O. Ulcerative colitis and colorectal cancer. N Engl J Med. 1990;323:1228–33. doi: 10.1056/NEJM199011013231802 2215606

[2] Eaden JA, Abrams KR, Mayberry JF. The risk of colorectal cancer in ulcerative colitis: a meta-analysis. Gut. 2001;48:526–35. doi: 10.1136/gut.48.4.526 11247898 PMC1728259

[3] Gillen CD, Andrews HA, Prior P, Allan RN. Crohn’s disease and colorectal cancer. Gut. 1994;35:651–5. doi: 10.1136/gut.35.5.651 8200559 PMC1374750

[4] Olén O, Erichsen R, Sachs MC, Pedersen L, Halfvarson J, Askling J, et al. Colorectal cancer in ulcerative colitis: a Scandinavian population-based cohort study. Lancet. 2020;395:123–31. doi: 10.1016/S0140-6736(19)32545-0 31929014

[5] Nieminen U, Jussila A, Nordling S, Mustonen H, Färkkilä MA. Inflammation and disease duration have a cumulative effect on the risk of dysplasia and carcinoma in IBD: a case-control observational study based on registry data. Int J Cancer J Int Cancer. 2014;134:189–96. doi: 10.1002/ijc.28346 23797639

[6] Gupta RB, Harpaz N, Itzkowitz S, Hossain S, Matula S, Kornbluth A, et al. Histologic inflammation is a risk factor for progression to colorectal neoplasia in ulcerative colitis: a cohort study. Gastroenterology. 2007;133:1099–105; quiz 1340–1. doi: 10.1053/j.gastro.2007.08.001 17919486 PMC2175077

[7] Jess T, Loftus EV, Jr, Velayos FS, Winther KV, Tremaine WJ, Zinsmeister AR, et al. Risk factors for colorectal neoplasia in inflammatory bowel disease: a nested case-control study from Copenhagen County, Denmark and Olmsted County, Minnesota. Am J Gastroenterol. 2007;102:829–36. doi: 10.1111/j.1572-0241.2007.01070.x 17222314

[8] Bonovas S, Fiorino G, Lytras T, Nikolopoulos G, Peyrin-Biroulet L, Danese S. Systematic review with meta-analysis: use of 5-aminosalicylates and risk of colorectal neoplasia in patients with inflammatory bowel disease. Aliment Pharmacol Ther. 2017;45:1179–92. doi: 10.1111/apt.14023 28261835

[9] Li H, Fan X, Houghton J. Tumor microenvironment: the role of the tumor stroma in cancer. J Cell Biochem. 2007;101:805–15. doi: 10.1002/jcb.21159 17226777

[10] D’Arcangelo E, Wu NC, Cadavid JL, McGuigan AP. The life cycle of cancer-associated fibroblasts within the tumour stroma and its importance in disease outcome. Br J Cancer. 2020;122:931–42. doi: 10.1038/s41416-019-0705-1 31992854 PMC7109057

[11] Fan S, Zhou L, Zhang W, Wang D, Tang D. Role of imbalanced gut microbiota in promoting CRC metastasis: from theory to clinical application. Cell Commun Signal. 2024;22:232. doi: 10.1186/s12964-024-01615-9 38637851 PMC11025274

[12] Lu W, Kang Y. Epithelial-mesenchymal plasticity in cancer progression and metastasis. Dev Cell. 2019;49:361–74. doi: 10.1016/j.devcel.2019.04.010 31063755 PMC6506183

[13] QUASAR Collaborative Group. Adjuvant chemotherapy versus observation in patients with colorectal cancer: a randomised study. Lancet. 2007;370:2020–9. doi: 10.1016/S0140-6736(07)61866-2 18083404

[14] AJCC cancer staging manual. Available from: https://link-springer-com.ezproxy.its.uu.se/book/9783319406176 [cited 10 January 2024].

[15] Xu M, Li Y, Liu Y, Chang J, Zhou C, Weng W, et al. The development and implementation of pathological parameters and molecular testing impact prognosis of colorectal adenocarcinoma. J Natl Cancer Cent. 2024;4:74–85. doi: 10.1016/j.jncc.2024.02.001 39036386 PMC11256523

[16] Yang L, Yang J, Kleppe A, Danielsen HE, Kerr DJ. Personalizing adjuvant therapy for patients with colorectal cancer. Nat Rev Clin Oncol. 2024;21:67–79. doi: 10.1038/s41571-023-00834-2 38001356

[17] Strous MTA, van der Linden RLA, Gubbels ALHM, Faes TKE, Bosscha K, Bronkhorst CM, et al. Node-negative colon cancer: histological, molecular, and stromal features predicting disease recurrence. Mol Med. 2023;29:77. doi: 10.1186/s10020-023-00677-8 37344790 PMC10283206

[18] Lea D, Håland S, Hagland HR, Søreide K. Accuracy of TNM staging in colorectal cancer: a review of current culprits, the modern role of morphology and stepping-stones for improvements in the molecular era. Scand J Gastroenterol. 2014;49:1153–63. doi: 10.3109/00365521.2014.950692 25144865

[19] Mesker WE, Junggeburt JMC, Szuhai K, de Heer P, Morreau H, Tanke HJ, et al. The carcinoma–stromal ratio of colon carcinoma is an independent factor for survival compared to lymph node status and tumor stage. Cell Oncol Off J Int Soc Cell Oncol. 2007;29:387–98. doi: 10.1155/2007/175276 PMC4617992 17726261

[20] Hansen TF, Kjær-Frifeldt S, Lindebjerg J, Rafaelsen SR, Jensen LH, Jakobsen A, et al. Tumor–stroma ratio predicts recurrence in patients with colon cancer treated with neoadjuvant chemotherapy. Acta Oncol. 2018;57:528–33. doi: 10.1080/0284186X.2017.1385841 28980848

[21] Park JH, Richards CH, McMillan DC, Horgan PG, Roxburgh CSD. The relationship between tumour stroma percentage, the tumour microenvironment and survival in patients with primary operable colorectal cancer. Ann Oncol. 2014;25:644–51. doi: 10.1093/annonc/mdt593 24458470 PMC4433525

[22] van Pelt GW, Sandberg TP, Morreau H, Gelderblom H, van Krieken JHJM, Tollenaar RAEM, et al. The tumour–stroma ratio in colon cancer: the biological role and its prognostic impact. Histopathology. 2018;73:197–206. doi: 10.1111/his.13489 29457843

[23] Polack M, Smit MA, van Pelt GW, Roodvoets AGH, Meershoek-Klein Kranenbarg E, Putter H, et al. Results from the UNITED study: a multicenter study validating the prognostic effect of the tumor–stroma ratio in colon cancer. ESMO Open. 2024;9:102988. doi: 10.1016/j.esmoop.2024.102988 38613913 PMC11033069

[24] Souza da Silva RM, Queiroga EM, Paz AR, Neves FFP, Cunha KS, Dias EP. Standardized assessment of the tumor-stroma ratio in colorectal cancer: interobserver validation and reproducibility of a potential prognostic factor. Clin Pathol. 2021;14:2632010X21989686. doi: 10.1177/2632010X21989686 PMC7887673 33634262

[25] Rutter M, Saunders B, Wilkinson K, Rumbles S, Schofield G, Kamm M, et al. Severity of inflammation is a risk factor for colorectal neoplasia in ulcerative colitis. Gastroenterology. 2004;126:451–9. doi: 10.1053/j.gastro.2003.11.010 14762782

[26] Saraggi D, Fassan M, Mescoli C, Scarpa M, Valeri N, Michielan A, et al. The molecular landscape of colitis-associated carcinogenesis. Dig Liver Dis. 2017;49:326–30. doi: 10.1016/j.dld.2016.12.011 28089111

[27] Guinney J, Dienstmann R, Wang X, de Reyniès A, Schlicker A, Soneson C, et al. The consensus molecular subtypes of colorectal cancer. Nat Med. 2015;21:1350–6. doi: 10.1038/nm.3967 26457759 PMC4636487

[28] Rajamäki K, Taira A, Katainen R, Välimäki N, Kuosmanen A, Plaketti R-M, et al. Genetic and epigenetic characteristics of inflammatory bowel disease-associated colorectal cancer. Gastroenterology. 2021;161:592–607. doi: 10.1053/j.gastro.2021.04.042 33930428

[29] Lutgens MWMD, van Oijen MGH, van der Heijden GJMG, Vleggaar FP, Siersema PD, Oldenburg B. Declining risk of colorectal cancer in inflammatory bowel disease: an updated meta-analysis of population-based cohort studies. Inflamm Bowel Dis. 2013;19:789–99. doi: 10.1097/MIB.0b013e31828029c0 23448792

[30] Olén O, Erichsen R, Sachs MC, Pedersen L, Halfvarson J, Askling J, et al. Colorectal cancer in Crohn’s disease: a Scandinavian population-based cohort study. Lancet Gastroenterol Hepatol. 2020;5:475–84. doi: 10.1016/S2468-1253(20)30005-4 32066530

[31] Everhov ÅH, Erichsen R, Järås J, Pedersen L, Halfvarson J, Askling J, et al. Colorectal cancer in elderly-onset inflammatory bowel disease: a 1969–2017 Scandinavian register-based cohort study. Aliment Pharmacol Ther. 2022;56:1168–82. doi: 10.1111/apt.17175 35916190 PMC9545052

[32] Sebastian S, Hernández V, Myrelid P, Kariv R, Tsianos E, Toruner M, et al. Colorectal cancer in inflammatory bowel disease: results of the 3rd ECCO pathogenesis scientific workshop (I). J Crohns Colitis. 2014;8:5–18. doi: 10.1016/j.crohns.2013.04.008 23664897

[33] Herrinton LJ, Liu L, Levin TR, Allison JE, Lewis JD, Velayos F. Incidence and mortality of colorectal adenocarcinoma in persons with inflammatory bowel disease from 1998 to 2010. Gastroenterology. 2012;143:382–9. doi: 10.1053/j.gastro.2012.04.054 22609382

[34] Lundqvist E, Westberg K, Boman SE, Myrberg IH, Everhov ÅH, Myrelid P, et al. Outcome after surgery for colon cancer in a national cohort of patients with and without inflammatory bowel disease. Aliment Pharmacol Ther. 2025;n/a:n/a. doi: 10.1111/apt.70296 PMC12646627 40696766

[35] Lundqvist E, Westberg K, Dietrich CE, Everhov ÅH, Myrelid P, Glimelius B, et al. Treatment and survival of non-metastatic rectal cancer in patients with inflammatory bowel disease: nationwide cohort study. BJS Open. 2025;9:zraf014. doi: 10.1093/bjsopen/zraf014 40131793 PMC11934924

[36] Bogach J, Pond G, Eskicioglu C, Seow H. Age-related survival differences in patients with inflammatory bowel disease – associated colorectal cancer: a population-based cohort study. Inflamm Bowel Dis. 2019;25:1957–65. doi: 10.1093/ibd/izz088 31066449

[37] Klimeck L, Heisser T, Hoffmeister M, Brenner H. Colorectal cancer: a health and economic problem. Best Pract Res Clin Gastroenterol. 2023;66:101839. doi: 10.1016/j.bpg.2023.101839 37852707

[38] Siegel RL, Wagle NS, Cercek A, Smith RA, Jemal A. Colorectal cancer statistics, 2023. CA Cancer J Clin. 2023;73:233–54. doi: 10.3322/caac.21772 36856579

[39] van Pelt GW, Kjær-Frifeldt S, van Krieken JHJM, Al Dieri R, Morreau H, Tollenaar RAEM, et al. Scoring the tumor-stroma ratio in colon cancer: procedure and recommendations. Virchows Arch. 2018;473:405–12. doi: 10.1007/s00428-018-2408-z 30030621 PMC6182321

[40] Therneau TM. A package for survival analysis in R. 2024. Available from: https://CRAN.R-project.org/package=survival [cited 28 October 2025].

[41] R Core Team. R: a language and environment for statistical computing. Vienna: R Foundation for Statistical Computing; 2024. Available from: https://www.R-project.org/ [cited 28 October 2025].

[42] Therneau TM, Grambsch PM. Modeling survival data: extending the cox model. New York, NY: Springer; 2000.

[43] Ishii G. New insights into cancer pathology learned from the dynamics of cancer-associated fibroblasts. Pathol Int. 2024;74:493–507. doi: 10.1111/pin.13461 38923250

[44] Feliu J, Gámez-Pozo A, Martínez-Pérez D, Pérez-Wert P, Matamala-Luengo D, Viñal D, et al. Functional proteomics of colon cancer consensus molecular subtypes. Br J Cancer. 2024;130:1670–8. doi: 10.1038/s41416-024-02650-6 38486123 PMC11091086

[45] Strous MTA, Faes TKE, Gubbels ALHM, van der Linden RLA, Mesker WE, Bosscha K, et al. A high tumour-stroma ratio (TSR) in colon tumours and its metastatic lymph nodes predicts poor cancer-free survival and chemo resistance. Clin Transl Oncol. 2022;24:1047–58. doi: 10.1007/s12094-021-02746-y 35064453

[46] Huijbers A, van Pelt GW, Kerr RS, Johnstone EC, Tollenaar RAEM, Kerr DJ, et al. The value of additional bevacizumab in patients with high-risk stroma-high colon cancer. A study within the QUASAR2 trial, an open-label randomized phase 3 trial. J Surg Oncol. 2018;117:1043–8. doi: 10.1002/jso.24998 29448309

[47] Cheddani H, Dauchet L, Fumery M, Charpentier C, Bouvier AM, Dupas J-L, et al. Cancer in elderly onset inflammatory bowel disease: a population-based study. Off J Am Coll Gastroenterol ACG. 2016;111:1428. doi: 10.1038/ajg.2016.304 27481308

[48] Zhu Y, Zhu M, Lance P. IL1β-mediated stromal COX-2 signaling mediates proliferation and invasiveness of colonic epithelial cancer cells. Exp Cell Res. 2012;318:2520–30. doi: 10.1016/j.yexcr.2012.07.021 22884582

[49] Zhu Y, Zhu M, Lance P. iNOS signaling interacts with COX-2 pathway in colonic fibroblasts. Exp Cell Res. 2012;318:2116–27. doi: 10.1016/j.yexcr.2012.05.027 22683859

[50] Björner K, Chen W-N, Gannavarapu VR, Axling F, Gulyas M, Halim MA, et al. High iNOS and IL-1β immunoreactivity are features of colitis-associated colorectal cancer tumors, but fail to predict 5-year survival. Ups J Med Sci. 2024;28:10241. doi: 10.48101/ujms.v128.10241 PMC10770641 38187473

[51] Li Y, Wang L, Pappan L, Galliher-Beckley A, Shi J. IL-1β promotes stemness and invasiveness of colon cancer cells through Zeb1 activation. Mol Cancer. 2012;11:87. doi: 10.1186/1476-4598-11-87 23174018 PMC3532073

[52] Sahoo K, Sundararajan V. IL-1β and associated molecules as prognostic biomarkers linked with immune cell infiltration in colorectal cancer: an integrated statistical and machine learning approach. Discov Oncol. 2025;16:252. doi: 10.1007/s12672-025-01989-3 40019680 PMC11871282

