# Supplementary material for: Tumor–stroma ratio in colitis-associated colorectal cancer
Source: Ups J Med Sci. 2026 Jan 2;130:10.48101/ujms.v130.13250. doi: 10.48101/ujms.v130.13250 (PMC12771069; doi:10.48101/ujms.v130.13250)
Supplement: Supplementary file 1 [file UJMS-130-13250-s1.pdf]

Copyright Dominic-Luc Webb 2025

Permission is granted to freely use this R code without notifying the author. This program processes data in similar manner as Monte Carlo Cross Validation. It randomly sub-samples survival data to create simulation datasets of varying sample size and calculates chi-square p-values. By default, 7 random sub-samplings from original dataset are generated, each with a separate p-value with no censoring. Censoring can be applied simply by unhashing the indicated two lines. The program then lists the resulting n and p-value in a single table for plotting, curve fitting etc. The output can be used to visualize deflection of p-values as sample size increases. If you find it helpful, please cite our paper: Björner K., Gulyas M., Hellström P. M., & Webb D.-L. (2026). Tumor-stroma ratio in colitis-associated colorectal cancer. Upsala Journal of Medical Sciences, 130, e13250. https://doi.org/10.48101/ujms.v130.13250

```
# Clear programming environment

rm (list = ls())


# Place data into arrays. First lines are original data. Subsequent lines are

# replicates (n=36 to n=360 for simulation purposes only)

# PID Patient code

# TSR Tumor stroma score (%)

# Surv Survival months

# Status 0 = default, 1 = event (e.g., death) occurred


PID <-
c(1,2,3,4,7,8,9,10,11,12,13,14,16,17,19,22,23,24,26,28,29,31,32,33,37,39,40,45,46,4
7,48,51,52,54,55,59,

1,2,3,4,7,8,9,10,11,12,13,14,16,17,19,22,23,24,26,28,29,31,32,33,37,39,40,45,46,47,
48,51,52,54,55,59,

1,2,3,4,7,8,9,10,11,12,13,14,16,17,19,22,23,24,26,28,29,31,32,33,37,39,40,45,46,47,
48,51,52,54,55,59,

1,2,3,4,7,8,9,10,11,12,13,14,16,17,19,22,23,24,26,28,29,31,32,33,37,39,40,45,46,47,
48,51,52,54,55,59,

1,2,3,4,7,8,9,10,11,12,13,14,16,17,19,22,23,24,26,28,29,31,32,33,37,39,40,45,46,47,
48,51,52,54,55,59,

1,2,3,4,7,8,9,10,11,12,13,14,16,17,19,22,23,24,26,28,29,31,32,33,37,39,40,45,46,47,
48,51,52,54,55,59,

1,2,3,4,7,8,9,10,11,12,13,14,16,17,19,22,23,24,26,28,29,31,32,33,37,39,40,45,46,47,
48,51,52,54,55,59,

1,2,3,4,7,8,9,10,11,12,13,14,16,17,19,22,23,24,26,28,29,31,32,33,37,39,40,45,46,47,
48,51,52,54,55,59,

1,2,3,4,7,8,9,10,11,12,13,14,16,17,19,22,23,24,26,28,29,31,32,33,37,39,40,45,46,47,
48,51,52,54,55,59,

1,2,3,4,7,8,9,10,11,12,13,14,16,17,19,22,23,24,26,28,29,31,32,33,37,39,40,45,46,47,
48,51,52,54,55,59)
```

```
TSR <-
c(60,60,10,70,60,30,60,70,70,90,40,60,70,80,80,30,80,60,60,20,20,60,70,80,70,30,20,
20,40,40,20,40,60,60,70,40,

60,60,10,70,60,30,60,70,70,90,40,60,70,80,80,30,80,60,60,20,20,60,70,80,70,30,20,20
,40,40,20,40,60,60,70,40,

60,60,10,70,60,30,60,70,70,90,40,60,70,80,80,30,80,60,60,20,20,60,70,80,70,30,20,20
,40,40,20,40,60,60,70,40,

60,60,10,70,60,30,60,70,70,90,40,60,70,80,80,30,80,60,60,20,20,60,70,80,70,30,20,20
,40,40,20,40,60,60,70,40,

60,60,10,70,60,30,60,70,70,90,40,60,70,80,80,30,80,60,60,20,20,60,70,80,70,30,20,20
,40,40,20,40,60,60,70,40,

60,60,10,70,60,30,60,70,70,90,40,60,70,80,80,30,80,60,60,20,20,60,70,80,70,30,20,20
,40,40,20,40,60,60,70,40,

60,60,10,70,60,30,60,70,70,90,40,60,70,80,80,30,80,60,60,20,20,60,70,80,70,30,20,20
,40,40,20,40,60,60,70,40,

60,60,10,70,60,30,60,70,70,90,40,60,70,80,80,30,80,60,60,20,20,60,70,80,70,30,20,20
,40,40,20,40,60,60,70,40,

60,60,10,70,60,30,60,70,70,90,40,60,70,80,80,30,80,60,60,20,20,60,70,80,70,30,20,20
,40,40,20,40,60,60,70,40,

60,60,10,70,60,30,60,70,70,90,40,60,70,80,80,30,80,60,60,20,20,60,70,80,70,30,20,20
,40,40,20,40,60,60,70,40)
```

```
Surv <-
c(6,5,2,7,11,19,23,13,35,15,24,29,25,29,46,59,120,150,240,222,240,300,276,20,126,75
,43,75,45,58,32,42,29,60,30,12,

6,5,2,7,11,19,23,13,35,15,24,29,25,29,46,59,120,150,240,222,240,300,276,20,126,75,4
3,75,45,58,32,42,29,60,30,12,

6,5,2,7,11,19,23,13,35,15,24,29,25,29,46,59,120,150,240,222,240,300,276,20,126,75,4
3,75,45,58,32,42,29,60,30,12,

6,5,2,7,11,19,23,13,35,15,24,29,25,29,46,59,120,150,240,222,240,300,276,20,126,75,4
3,75,45,58,32,42,29,60,30,12,

6,5,2,7,11,19,23,13,35,15,24,29,25,29,46,59,120,150,240,222,240,300,276,20,126,75,4
3,75,45,58,32,42,29,60,30,12,

6,5,2,7,11,19,23,13,35,15,24,29,25,29,46,59,120,150,240,222,240,300,276,20,126,75,4
3,75,45,58,32,42,29,60,30,12,

6,5,2,7,11,19,23,13,35,15,24,29,25,29,46,59,120,150,240,222,240,300,276,20,126,75,4
3,75,45,58,32,42,29,60,30,12,

6,5,2,7,11,19,23,13,35,15,24,29,25,29,46,59,120,150,240,222,240,300,276,20,126,75,4
3,75,45,58,32,42,29,60,30,12,

6,5,2,7,11,19,23,13,35,15,24,29,25,29,46,59,120,150,240,222,240,300,276,20,126,75,4
3,75,45,58,32,42,29,60,30,12,

6,5,2,7,11,19,23,13,35,15,24,29,25,29,46,59,120,150,240,222,240,300,276,20,126,75,4
3,75,45,58,32,42,29,60,30,12)
```

```
Status <-
c(1,1,1,1,1,1,1,1,1,1,1,1,1,1,1,0,0,0,0,0,0,0,0,1,0,1,0,0,0,0,1,0,1,0,1,0,
1,1,1,1,1,1,1,1,1,1,1,1,1,1,1,0,0,0,0,0,0,0,0,1,0,1,0,0,0,0,1,0,1,0,1,0,
1,1,1,1,1,1,1,1,1,1,1,1,1,1,1,0,0,0,0,0,0,0,0,1,0,1,0,0,0,0,1,0,1,0,1,0,
1,1,1,1,1,1,1,1,1,1,1,1,1,1,1,0,0,0,0,0,0,0,0,1,0,1,0,0,0,0,1,0,1,0,1,0,
1,1,1,1,1,1,1,1,1,1,1,1,1,1,1,0,0,0,0,0,0,0,0,1,0,1,0,0,0,0,1,0,1,0,1,0,
1,1,1,1,1,1,1,1,1,1,1,1,1,1,1,0,0,0,0,0,0,0,0,1,0,1,0,0,0,0,1,0,1,0,1,0,
1,1,1,1,1,1,1,1,1,1,1,1,1,1,1,0,0,0,0,0,0,0,0,1,0,1,0,0,0,0,1,0,1,0,1,0,
1,1,1,1,1,1,1,1,1,1,1,1,1,1,1,0,0,0,0,0,0,0,0,1,0,1,0,0,0,0,1,0,1,0,1,0,
1,1,1,1,1,1,1,1,1,1,1,1,1,1,1,0,0,0,0,0,0,0,0,1,0,1,0,0,0,0,1,0,1,0,1,0,
1,1,1,1,1,1,1,1,1,1,1,1,1,1,1,0,0,0,0,0,0,0,0,1,0,1,0,0,0,0,1,0,1,0,1,0)

# Place data into data frame
all_TSR <- data.frame (
PID = PID,
TSR = TSR,
Surv = Surv,
Status = Status
)

# Load survival library and ensure ability to print all data
library(survival)
options(max.print = 1440)

# Ensure N_pval array is clear prior to any new run
rm(N_pval)

# Fill array, starting with n=6, in increments of 3 until n=36.
min_sample = 6
for (n in seq(from = min_sample, to = 36, by = 3)) {

# Collect 7 p-values per n.
for (i in 1:7) {
sample_indices <- sample(1:nrow(all_TSR), n, replace = FALSE)
subgroup <- all_TSR[sample_indices, ]

# Unhash these two lines to perform censoring (currently set to 60 months)
```

```
# subgroup$Surv <- pmin(subgroup$Surv, 60)
# subgroup$Status[subgroup$Surv == 60] <- 0

subgroup$high_low <- ifelse(subgroup$TSR > 50, "High_TSR", "Low_TSR")
subgroup$high_low <- factor(subgroup$high_low, levels = c("Low_TSR", "High_TSR"))

result <- tryCatch({
surv_obj <- Surv(time = subgroup$Surv, event = subgroup$Status)
test_result <- survdiff(surv_obj ~ high_low, data = subgroup)
  }, error = function(e) {
  if (grepl("There is only 1 group", e$message)) {
    message("Skipping run: only 1 group present.")
    return(NULL)  # return NULL or a default value to indicate skip
  } else {
    stop(e)  # rethrow other errors
  }
})

subgroup
test_result
test_result$pvalue

if (!exists("N_pval")) {
  N_pval <- data.frame(n = integer(), pvalue = numeric())
}
# Each run adds a new row
N_pval <- rbind(N_pval, data.frame(n = n, pvalue = test_result$pvalue))
}
}

# list n and p-values. Copy to ploting software. If Excel, Use text to number.
# In OpenOffice, click space and tab delimiters and merge.
print(N_pval)
```
